# Coexistence of treatment-related *MLL* cleavage and rearrangement in a child with haemophagocytic lymphohistiocytosis

**DOI:** 10.1038/sj.bjc.6602269

**Published:** 2004-11-30

**Authors:** A Ng, P F Ravetto, G M Taylor, R F Wynn, O B Eden

**Affiliations:** 1 Immunogenetics Laboratory, University of Manchester, Central Manchester and Manchester Children's University Hospitals Trust, Manchester, UK; 2 Academic Unit of Paediatric Oncology, Central Manchester and Manchester Children's University Hospitals Trust, Manchester, UK; 3 Department of Paediatric Haematology, Central Manchester and Manchester Children's University Hospitals Trust, Manchester, UK

**Keywords:** treatment-related leukaemia, *MLL* cleavage and rearrangement, HLH

## Abstract

Treatment-related acute myeloid leukaemia (t-AML) is a serious complication of topoisomerase 2 inhibitor therapy and is characterised by the presence of mixed lineage leukaemia (*MLL*) rearrangement. By molecular tracking, we were able to show that *MLL* cleavage preceded gene rearrangement by 3 months and before the clinical diagnosis of t-AML in a patient with haemophagocytic lymphohistiocytosis. This is the first report on the sequential detection of the two biomarkers in treatment-related leukaemogenesis.

The relationship between topoisomerase 2 (topo 2) inhibitor therapy and the pathogenesis of treatment-related acute myeloid leukaemia (t-AML) with rearrangements of the mixed lineage leukaemia (*MLL*) gene is now well documented (Felix, 1998; Pui and Relling, 2002). This type of t-AML tends to have a myelomonocytic/monoblastic phenotype and rearrangements of the *MLL* gene with multiple partners. Cytogenetic analysis has revealed a median latency from primary disease to t-AML of 24 months (range 10–100 months). In addition to its clinical importance as an adverse outcome of chemotherapy, t-AML provides an opportunity to investigate the timing of the molecular events involved in the pathogenesis of this condition in relation to the administration of topo 2 inhibitor therapy. We have backtracked the molecular events from a t-AML in a child with Epstein–Barr virus (EBV)-related haemophagocytic lymphohistiocytosis (HLH).

## PATIENT AND METHOD

The patient was an 18-month-old boy, diagnosed with EBV-related HLH, and treated according to the HLH-94 protocol (Henter *et al*, 1997) with dexamethasone, intrathecal methotrexate and intravenous etoposide (150 mg m^−2^) given twice weekly in the first 2 weeks and then weekly for 5 weeks (cumulative etoposide dose: 1.35 g m^−2^). At 2 months after the start of treatment, the patient was in complete remission, but by the 6th month after HLH diagnosis (4 months after treatment ceased), he presented with jaw swelling and cervical lymphadenopathy. Bone marrow examination revealed 70% infiltration with myeloid blasts, confirmed by cytochemistry and immunophenotyping as monoblastic AML. There was no CNS involvement by leukaemia. Interphase cytogenetics of the bone marrow showed a chromosome 9;11 translocation (47, XY, +8, t(9;11)(p22;q23)). The patient received induction chemotherapy (daunorubicin, cytarabine and thioguanine) for his AML according to the MRC 10th AML trial (Stevens *et al*, 1998) and achieved remission. At 5 months after the diagnosis of t-AML, he was successfully transplanted with non-T-cell-depleted bone marrow from an HLA-matched unrelated donor, following conditioning with cyclophosphamide and total body irradiation. He remains in complete remission 28 months post-transplantation.

We collected serial blood and bone marrow samples at the time of the patient's HLH diagnosis, and thereafter at weekly intervals for 6 weeks, and then again at 3 and 6 months. Prior approval for the study was obtained from the local research ethics committee. Genomic DNA extracted from immediately frozen samples was digested with *Bam*H1 and analysed for etoposide-induced *MLL* cleavage (Aplan *et al*, 1996) and rearrangement, using a 0.74 kb cDNA probe spanning the *MLL* breakpoint cluster region (BCR). The DNA was size fractionated by 0.7% agarose gel electrophoresis, and blotted onto nylon membranes. *MLL* cleavage was detected, after hybridisation overnight at 60°C with the ^32^P-labelled *MLL* probe, using real-time autoradiography. Panhandle PCR method (Felix and Jones, 1998) was also used to analyse the same serial blood and bone marrow samples for *MLL* rearrangement.

## RESULTS

**Figure 1:**
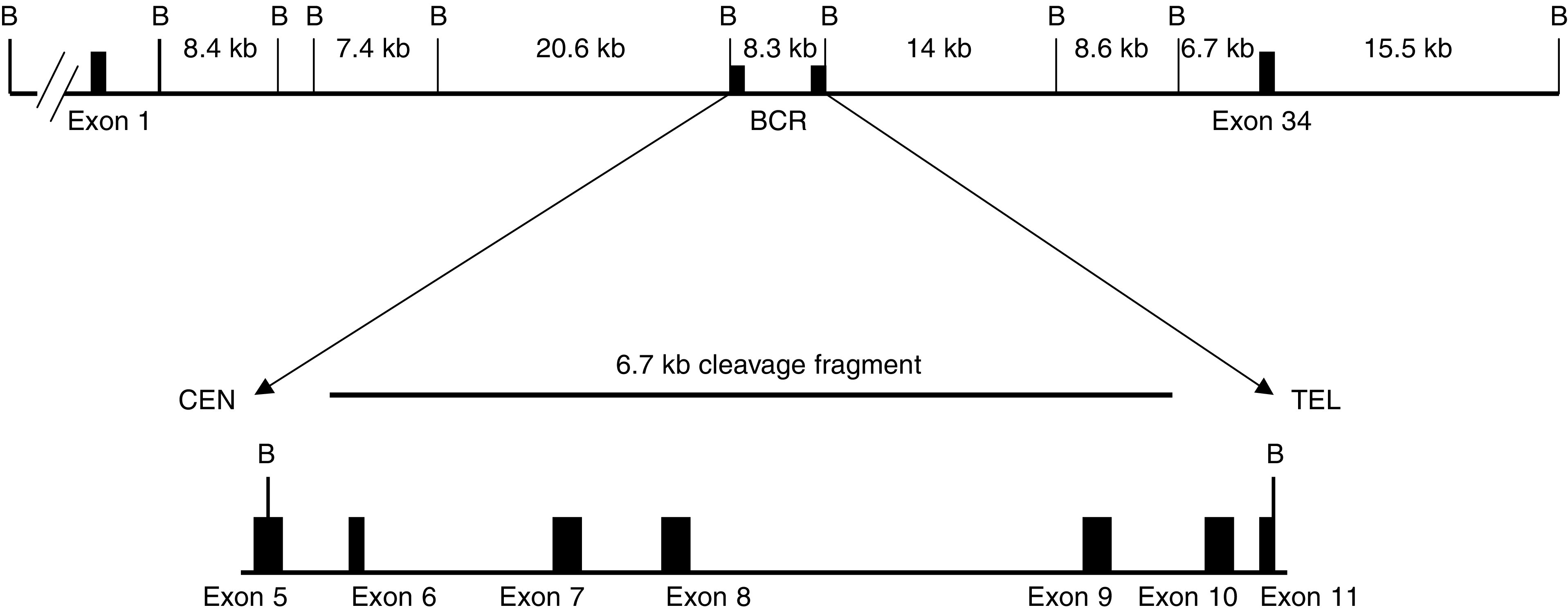
Human genomic *MLL* with BCR and *MLL* cleavage fragment. Schematic representation of the genomic *MLL* locus with *Bam*H1 restriction enzyme sites, the BCR and the etoposide-induced *MLL* cleavage fragment in this patient. B – *Bam*H1 sites.

We detected no *MLL* cleavage in any of the peripheral blood samples obtained up to the appearance of overt t-AML, 6 months after the diagnosis of HLH. However, a 6.7 kb *MLL* cleavage fragment, identical to that detected when we incubated the haemopoietic cell line BV173 *in vitro* with 10 *μ*M etoposide for 6 h, was detected in the bone marrow (morphologically <5% blasts and no circulating blast seen) obtained 3 months after HLH diagnosis, 1 month after the last dose of etoposide (cumulative dose: 1.35 g m^−2^) and 3 months prior to the diagnosis of overt t-AML. Analysis of Southern blots of the same *Bam*H1-digested DNA samples revealed two rearranged *MLL* fragments (5.6, 18 kb), in addition to the 8.3 kb germline *MLL* fragment, only in the bone marrow obtained at the time of t-AML diagnosis (Figures 1

and 2

**Figure 2:**
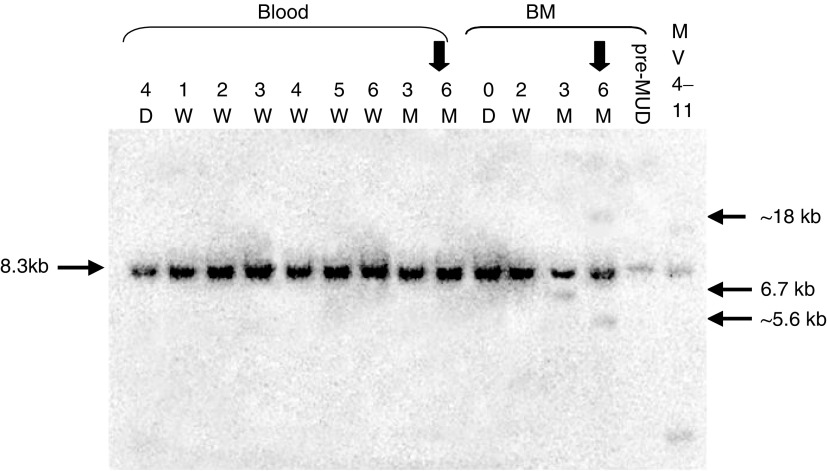
Southern blot analysis of serial samples for *MLL* cleavage and rearrangement. InstantImager bitmap showing the detection of ^32^P-labelled *MLL* probe hybridisation. *MLL* cleavage fragment (6.7 kb) was identified in the bone marrow 3 months after the start of etoposide therapy. *MLL* rearranged fragments (5.6, 18 kb) and germline BCR fragment (8.3 kb) were only detected at the clinical presentation of t-AML. *MLL* rearrangement resolved with further chemotherapy prior to the bone marrow transplant. D – day; W – week; M – month, from the diagnosis of HLH; ↓ – clinical presentation of t-AML; BM – bone marrow; MUD – matched, unrelated donor transplant.

). Panhandle PCR confirmed the presence of *MLL* rearrangement and the 5.6 kb der (11) *MLL* PCR product was only detected in the same bone marrow sample (Figure 3

**Figure 3:**
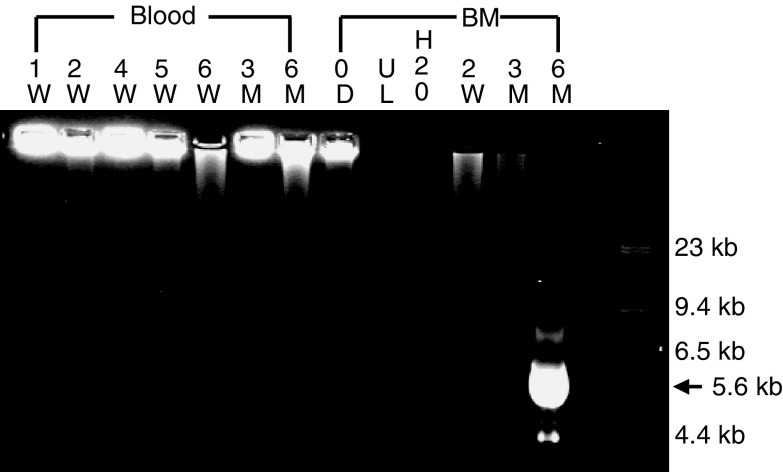
Panhandle PCR on the same samples for the detection of *MLL* rearrangement. Gel doc image showing the panhandle PCR product (5.6 kb) after agarose gel electrophoresis. It was only detected in the patient's bone marrow 6 months after the start of etoposide therapy. Negative controls: UL – unligated, H_2_O – water; BM – bone marrow; D – day; W – week; M – month, from the diagnosis of HLH.

). This was 4 months after HLH therapy was stopped, and 3 months after the detection of *MLL* cleavage in the bone marrow.

## DISCUSSION

The occurrence of t-AML involving *MLL* rearrangements with a latent period of 24–36 months has previously been reported in HLH (Takahashi *et al*, 1998; Kitazawa *et al*, 2001). Based on the clinical evidence alone, the latent period from the start of therapy until the diagnosis of overt t-AML in our patient was only 6 months. However, using molecular tracking, we have shown prospectively that etoposide-induced *MLL* cleavage preceded *MLL* rearrangement by 3 months, and furthermore, using the panhandle PCR method, that *MLL* rearrangement could only be detected at the diagnosis of t-AML. As no DNA samples were available to us in the time period between 3 and 6 months after HLH diagnosis, our conservative estimate is that the t-AML clone emerged during a period ⩽3 months. Cytogenetic and provisional sequencing data conclude that the rearrangement involved *AF9* as the partner gene, with nucleotides insertion within the breakpoint junction (intron 6 of *MLL*, intron 4 of *AF9*) of the fusion gene.

Our results differ from those reported by Megonigal *et al* (2000), who detected a leukaemic clone with *MLL-GAS7* within 6 weeks of the start of neuroblastoma therapy including doxorubicin. This was some 15.5 months before the appearance of clinically overt t-AML. The result that we obtained suggests that once established, the putative *MLL-AF9* leukaemic clone developed much more rapidly into overt AML as compared to the *MLL-GAS7* clone. It also suggests that partner sequences in *MLL* fusion genes could modify the leukaemogenic process.

As far as we are aware, this is the first case of t-AML in which *MLL* cleavage and rearrangement have been detected consecutively in a patient developing t-AML. Our finding thus suggests that the two are temporally related, with gene cleavage and DNA processing prior to translocation, as proposed in the work by Lovett *et al* (2001).

## References

[bib1] Aplan PD, Chervinsky DS, Stanulla M, Burhans WC (1996) Site-specific DNA cleavage within the *MLL* breakpoint cluster region induced by topoisomerase 2 inhibitors. Blood 87: 2649–2658 8639880

[bib2] Felix CA (1998) Secondary leukaemias induced by topoisomerase-targeted drugs. Biochim Biophys Acta 1400: 233–255 9748598 10.1016/s0167-4781(98)00139-0

[bib3] Felix CA, Jones DH (1998) Panhandle PCR: a technical advance to amplify *MLL* genomic translocation breakpoints. Leukemia 12: 976–981 9639429 10.1038/sj.leu.2401026

[bib4] Henter JI, Arico M, Egeler RM, Elinder G, Favera BE, Filipovich AH, Gadner H, Imashuku S, Janka-Schaub G, Komp D, Ladisch S, Web D (1997) HLH-94: a treatment protocol for hemophagocytic lymphohistiocytosis. Med Pediatr Oncol 28: 342–347 9121398 10.1002/(sici)1096-911x(199705)28:5<342::aid-mpo3>3.0.co;2-h

[bib5] Kitazawa J, Ito E, Arai K, Yokoyama M, Fukayama M, Imashuku S (2001) Secondary acute myelocytic leukemia after successful chemotherapy with etoposide for Epstein–Barr virus-associated hemophagocytic lymphohistiocytosis. Med Pediatr Oncol 37: 153–154 11496359 10.1002/mpo.1189

[bib6] Lovett BD, Nigro LL, Rappaport EF, Blair IA, Osheroff N, Zheng N, Megonigal MD, Williams WR, Nowell PC, Felix CA (2001) Near-precise interchromosomal recombination and functional DNA topoisomerase 2 cleavage sites at *MLL* and *AF-4* genomic breakpoints in treatment-related acute lymphoblastic leukemia with t(4;11) translocation. Proc Natl Acad Sci USA 98: 9802–9807 11493704 10.1073/pnas.171309898 PMC55533

[bib7] Megonigal MD, Cheung N-KV, Rappaport EF, Nowell PC, Wilson RB, Jones DH, Addya K, Leonard DGB, Kushner BH, Williams TM, Lange BJ, Felix CA (2000) Detection of leukaemia-associated *MLL-GAS7* translocation early during chemotherapy with DNA topoisomerase 2 inhibitors. Proc Natl Acad Sci USA 97: 2814–2819 10706619 10.1073/pnas.050397097 PMC16012

[bib8] Pui CH, Relling MV (2002) Topoisomerase 2 inhibitor-related acute myeloid leukaemia. Br J Haematol 109: 13–23 10.1046/j.1365-2141.2000.01843.x 10848777

[bib9] Stevens RF, Hann IM, Wheatley K, Gray RG (1998) Marked improvements in outcome with chemotherapy alone in paediatric acute myeloid leukaemia: results of the United Kingdom Medical Research Council's 10th AML trial. MRC Childhood Leukaemia Working Party. Br J Haematol 101: 130–140 9576193 10.1046/j.1365-2141.1998.00677.x

[bib10] Takahashi T, Yagasaki F, Endo K, Takahashi M, Itoh Y, Kawai N, Kusumoto S, Murohashi I, Bessho M, Hirashima K (1998) Therapy-related AML after successful chemotherapy with low dose etoposide for virus-associated hemophagocytic syndrome. Int J Hematol 68: 333–336 9846019 10.1016/s0925-5710(98)00070-x

